# Eco-Fabrication of Rigid Lignofoams with Porous Cellular Channels Coated by Polypropylene Films for Thermal Insulation Materials

**DOI:** 10.3390/polym18050548

**Published:** 2026-02-25

**Authors:** Qiangu Yan, Neda Arabzadeh Nosratabad, Timothy Ketelboeter, Craig Clemons, Liu Liu, Caixia Wan, Peter Kitin, Zhiyong Cai

**Affiliations:** 1 Forest Products Laboratory, USDA Forest Service, One Gifford Pinchot Drive, Madison, WI 53726, USA; 2 Department of Chemical and Biomedical Engineering, University of Missouri, 1406 Rollins Street, Columbia, MO 65211, USA

**Keywords:** lignin, biocomposite, self-expanding foam, rigid foam, polypropylene films, thermal insulation materials

## Abstract

This paper introduced a simple, efficient method to prepare mechanically strong lignin-based foams (lignofoams) with open-cell structures using a facile baking technique. The self-expansion of lignin occurred without any additional chemical blowing agents, foaming agents, plasticizers, or lubricants. During heating, kraft lignin softened, and the internal water, either initially adsorbed or generated in situ through the dehydration of hydroxyl groups, acted as a natural blowing agent for foaming a porous foam structure. Incorporating a small amount of polypropylene (PP) enhanced mechanical properties by coating the inner walls of open cells. The porous, softened composite was then cooled to room temperature and solidified into the self-expanded lignofoam. The resulting lignofoams exhibited tunable densities ranging from 0.21 to 0.49 g/cm^3^ and a maximum compressive strength of 3.6 MPa. The lignofoam also showed excellent thermal insulation properties with low thermal conductive coefficients (0.057–0.098 W/mK). These features highlight the great potential of lignofoam for a bio-based thermal insulation material for construction applications.

## 1. Introduction

Foams can be classified into two categories: non-self-expanding and self-expanding foam. Non-self-expanding foams are most commonly used and are typically produced through complex formulations involving polymers, chemical precursors, catalysts, blowing agents, surfactants, or crosslinking agents, depending on specific formulation [1,2]. In contrast, self-expanding foams can be produced through simplified processes, where either internal moisture or thermally self-decomposing groups vaporize during heating, generating the pressure needed to form a porous structure without the use of added chemical activators or blowing agents [3,4]. These mechanisms not only ease processing but also address the environmental and safety concerns associated with synthetic additives. Commercially available foams are mainly made from synthetic petroleum-based polymers such as polyurethane (PU), polystyrene (PS), or polyvinyl chloride (PVC), most of which are not self-expanding foams and are formed using chemical blowing agents and additives for structure formation [5,6,7]. These synthetic foams pose significant environmental challenges including non-biodegradability, reliance on petroleum sources, and increased pollution and ecological concerns over recycling and the disposal of post-consumer foams [7,8]. In this context, developing bio-based, self-expanding foams offers a promising route toward sustainable and simplified alternatives to conventional petroleum-based foams.

Bio-based polymer products offer sustainable alternatives to replacing conventional non-biodegradable petroleum-based polymers [9,10]. These biodegradable polymers, made from renewable resources, such as lignin, cellulose, and other natural precursors, have gained considerable attention in recent years. Lignin is the most abundant naturally occurring aromatic biopolymer, and its principle biological functions include mechanical support, structural integrity and aiding water transport within plants [11,12]. From the perspective of lignocellulosic biorefining, lignin valorization into value-added products is considered crucial for improving the profitability of biorefineries, although current paper and pulp mills limit lignin’s use in combustion for heat and power supply [7,13]. Lignin has been explored for the fabrication of various foam products with most involving chemically modified lignin, lignin-derived oligomers/monomers, and lignin as a crosslinker. Typically, such lignin-based foams incorporate less than 40% lignin [2,10], and their fabrication methods are conventional, involving labor/energy-consuming steps and the use of hazardous chemicals. Therefore, innovative techniques should be developed for lignin foam fabrication that enable simple, scalable, eco-friendly manufacturing. To this end, we pioneered eco-foaming based on a baking method for making self-expanding lignin foam (so-called lignofoam). In such a foaming process, inherent water (either adsorbed in raw lignin or generated in situ from the dehydration of hydroxyl groups) acted as a natural blowing agent, significantly regulating foam formation and structural characteristics [14]. The resulting lignofoams exhibited satisfying mechanical and thermal insulation properties given lignin as the sole precursor. The eco-foaming technique can be extended to lignin composite foams such as the incorporation of synthetic plastics with tailored properties for downstream processing. Overall, this innovative technique has opened a new avenue to the manufacturing of self-expanding lignin-based foams. Lignin is naturally brittle and rigid due to its complex, irregular aromatic structure and limited capacity for molecular entanglement. The unmodified lignin makes the foam brittle, friable, and prone to reduced tensile strength. The aromatic structure increases hard segments, causing increased brittleness and lower toughness [15]. On the other hand, polypropylene (PP) is a thermoplastic known for high tensile strength combined with excellent fatigue resistance and toughness [16].

The main objective of this paper was to eco-foam lignin composite foams by incorporating polypropylene (PP) as an additive for tailored properties. The effects of PP addition and foaming parameter (especially temperature) on the foam properties were studied. The resulting foams were characterized mainly for open-cell structures, mechanical strength, and thermal insulation properties. The role of PP in lignofoam structure and mechanical and thermal properties were revealed, and the mechanisms involving concurrent foaming and coating was proposed. This work demonstrated the facile fabrication of self-expanding lignin composite foams with property tailoring. It would present a significant advancement toward the mass production of low-cost, durable bio-based foams for construction and insulation applications.

## 2. Materials and Methods

### 2.1. Materials

Kraft lignin (BioChoice) was supplied by Domtar Corp. (Plymouth, NC, USA). The lignin was dried in air at room temperature and reached 7.4% moisture. PP flake (Bapolene^®^4012F) was obtained from Bamberger Polymers, Inc. (Jericho, NY, USA) and had a diameter of about 0.1–1 mm.

### 2.2. Foam Fabrication

Lignofoams were fabricated by a baking process [14] from kraft lignin mixed with PP at varying ratios (0–30% PP) through molding while maintaining a total mass of 300 g (Table 1). In brief, the kraft lignin–PP mixture was homogenized using a multi-speed blender (Hamilton Beach 50128 10 Function Blender, Hamilton Beach Brands, Inc. Glen Allen, VA, USA) for 10 min to ensure the uniform dispersion of PP in the mixtures, facilitating structural consistency in the final foam. The blended mixture was cold pressed in a mold at 0.5 MPa, forming a dense preform (referred to as PPL). The preformed PPL was then transferred to a larger mold to allow for expansion. The mold was then heated in an oven at a controlled rate of 10 °C/min up to 240 °C [14]. The temperature was maintained for 60 min under a constant low pressure of 100 Pa. During heating, lignin softened, and inherent water vaporized and was turned into a natural blowing agent. Following heating, the foamed material was gradually cooled down to room temperature, solidifying the expanded foam structure and stabilizing the open-cell morphology. The resulting self-expanded, open-celled lignofoam (PPLF) was removed from the mold and characterized for mechanical and thermal insulation properties.

### 2.3. Characterization Techniques

The thermal characteristics of kraft lignin were analyzed by thermogravimetric analysis (TGA) and differential scanning calorimetry (DSC) using a Mettler–Toledo TGA/DSC3^+^ system (Mettler-Toledo). Samples (20–30 mg) were heated from 30 to 240 °C with a ramp rate of 10 °C/min under a nitrogen flow (50 mL/min). The chemical structure of kraft lignin and PPL preforms was probed by attenuated total reflectance Fourier-transform infrared (ATR-FTIR) spectroscopy using a Thermo iZ10 spectrometer (Thermo Scientific, Nicolet iZ10,Thermo Fisher, Waltham, MA, USA) at a resolution of 4 cm^−1^ over 128 scans from 650 to 4000 cm^−1^. The expansion rates of the PPLFs were calculated by dividing the density of the cold-pressed PPL precursors by the density of the cured PPLFs. The foam samples were imaged by scanning electron microscopy using a Zeiss Gemini 450 field emission scanning electron microscope (Carl Zeiss AG, JEOL USA, Inc., Peabody, MA, USA) operated at 5 mm working distance and 5 kV accelerating voltage. The foams were cryo-fractured after freezing with liquid nitrogen, mounted on conductive carbon tape, and sputter coated with gold. Compression tests of foams were performed on 25.4 × 25.4 ×12.5 mm according to ASTM C365 using a 5869 Universal Testing Machine (Instron, Norwood, MA, USA) [17,18]. Samples were conditioned at 23 °C and 65% relative humidity for 2 weeks before testing. Thermal conductivity was measured using a Hot Disk TPS 1500 analyzer with a Kapton 8563 sensor (ThermTest, Fredericton, NB, Canada) following the ISO/DIS 22007-2.2 standard [14]. All samples were conditioned at 23 ± 1 °C and 65 ± 2% relative humidity (RH) until their weight was stabilized (approximately two weeks) before testing. Three replicates of each formulation were tested, and the average values and standard deviations were reported.

## 3. Results and Discussion

### 3.1. Effects of PP Content and Temperature on Chemical Structure and Thermal Properties

**Figure 1:**
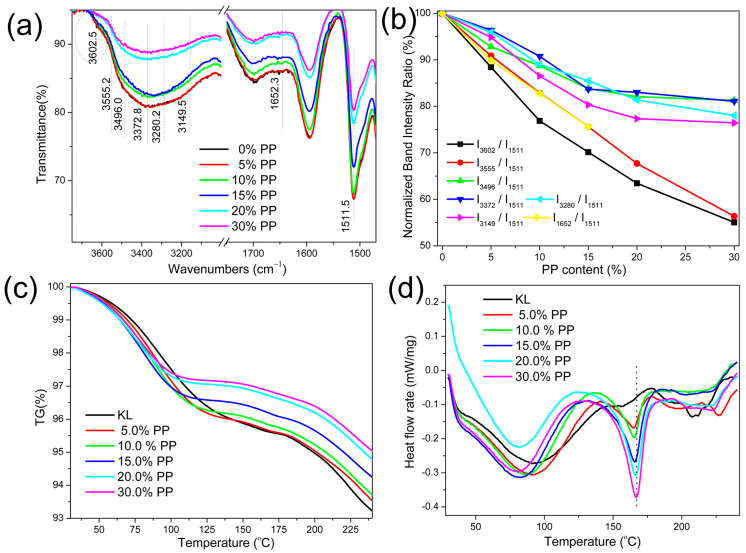
Effect of PP content on the chemical structure and thermal properties of PPL. (**a**) FTIR spectra, (**b**) normalization of intensity ratios of adsorbed water and –OH group bands to the aromatic skeleton band (1511.5 cm^−1^), (**c**) TGA curves, (**d**) DSC curves.

To investigate the effect of PP on the lignofoaming process, 0–30 wt% PP was blended with kraft lignin. The FTIR spectra of the pressed composite preforms (PPLs) are shown in Figure 1a. Bands assigned to lignin (e.g., 3700 to 3000 cm^−1^ corresponding to –OH groups) are apparent. However, FTIR bands for PP (Table S2) were not found. This was likely due to the fact that relatively small amounts of PP were added, and the PP was present as relatively large (0.1–1 mm diameter), discrete particles distributed in the lignin. Consequently, the odds of a particle appearing within the interaction volume of the beam were inconsistent at best. This was not an issue for the lignin particles, which were much smaller (0.1–100 µm) and existed as the majority phase.

Nevertheless, FTIR spectroscopy showed the influence of PP on the lignin fraction even though PP itself was not detected. This might be due to PP negatively affecting the compression of the preform and, consequently, limiting hydrogen bonding between lignin particles. Bands due to –OH group vibrations are identified in Figure 1a. Their intensities relative to the aromatic skeleton band (1511.5 cm^−1^) were calculated, normalized, and plotted in Figure 1b. Increasing PP contents from 0 to 30% resulted in decreasing –OH band intensities. Interestingly, the intensities of the –OH bands that are affected by adsorbed water (3602.5, 3555.2, and 1652.3 cm^−1^) attenuated faster than those of other hydroxyl groups (3496.0, 3372.8, 3280.2, and 3149.5 cm^−1^).

The TGA curves for the PPLs are shown in Figure 1c. Three stages of weight loss between 30 and 240 °C can be seen. The temperature ranges, peak temperature, and weight losses of each stage are listed in Table S1. The first stage is characterized by weight loss due to the evaporation of adsorbed moisture. As the PP content is increased, the adsorbed moisture decreases proportionally from 4.06 to 2.83%, which is likely due to the hydrophobic nature of PP, which limits water retention in the lignin matrix. Also, the adsorbed moisture is lost more quickly, which is possibly due to structural changes induced by PP that affected the distribution of water molecules and their release during heating.

The second weight loss stage is mainly attributed to the removal of chemically bound water and dehydration reactions involving the hydroxyl groups of lignin. For example, others have shown that the –OH group band is attenuated significantly due to dehydration reactions when lignin is heated to 200 °C [4,14], while other functional groups remain unchanged. The TGA mass loss peaks become wider, and the peak temperatures shift to higher temperatures with increasing PP contents in the PPLs (Figure 1c and Table S1). The mass losses of this step are relatively low and increase slightly with the increase in PP content from 0.42% for PPL1 (0% PP) to 0.66% for PPL6 (30% PP). PPLs with PP contents of 5%, 10%, and 15% (PPL2, PPL3 and PPL4) show very similar behavior.

The third weight loss stage is mainly attributed to the release of trapped steam bubbles in the lignin matrix, which softens considerably at the elevated temperatures of this stage. The steam generated from the dehydration reactions serves as the blowing agent. With increasing temperature, more hydroxyl groups dehydrate and form steam, causing continued bubble expansion in the softened lignin. When the steam pressure in the bubbles is high enough, it can rupture the softened lignin walls and generate an interconnected foam structure as some steam escapes. For the sole lignin sample, a weight loss of 2.31% occurs between 178.4 and 240 °C. By increasing the PP content, the onset of weight loss is delayed (i.e., shifts to higher temperatures), and less weight is lost (Table S1). The latter is partly due to the fact that PP is stable at 240 °C, and there is less lignin to generate volatile products. However, the weight losses exceed what would be expected from the dilution effect alone, and they may also be due to continued condensation reactions or differences related to foam structure.

The PPLs were also analyzed using DSC to further understand the behavior in the three temperature ranges described above. The results are plotted in Figure 1d. The first stage (30–146 °C) shows an endotherm partially due to the evaporation of adsorbed moisture but also due to molecular relaxation as a result of the prior thermal history of the lignin [19]. While the second stage (146–178.4 °C) includes the release of chemically bound water, heat flow related to lignin condensation, the onset of lignin softening, etc., the DSC curve was dominated by the melting endotherm of PP when PP was added. PP is a semicrystalline material, and this melting endotherm represents a rapid transition from a solid to molten polymer. Conversely, kraft lignin is an amorphous polymer and has a glass transition of about 148 °C [20]. As with many amorphous polymers, the softening of lignin occurs over a wide temperature range, and it does not flow significantly until it is heated well above its glass transition temperature. In the third temperature range (178.4–240 °C), a complicated mix of events such as the continued softening and dehydration of lignin and steam formation and escape influenced the heat flow curves.

In order to determine the thermal stability and structural stability of the lignofoam samples, TGA and DSC analyses were performed under four successive heating cycles by comparing kraft lignin with 15% PPLF foam (Figure S1a–f). In the first heating cycle, kraft lignin exhibited significant mass loss below 100 °C, primarily due to moisture evaporation and volatile release, while the 15% PPLF foam displayed lower initial mass loss, indicating reduced moisture retention due to the presence of PP (Figure S1a,d). DTG curves confirmed this observation, with kraft lignin showing a distinct sharp peak at the low temperature range, whereas the foam had more of a gradual decomposition characteristic (Figure S1b,e). This indicates that the incorporation of PP influences thermal behavior by restricting moisture uptake and stabilizing the foam structure. The DSC results further supported these findings, wherein kraft lignin had a pronounced endothermic peak in the first cycle, corresponding to water removal and lignin thermal relaxation, while the PP-containing foam exhibited a more controlled and stable thermal response (Figure S1c,f). After the first cycle, both materials reached a thermal stabilization state, with minor mass losses and variations in heat flow during cycles 2–4, confirming that significant thermal events only occur during the first heating. PP presence also delayed thermal degradation onset in the foam, exhibiting higher thermal stability than the sole lignin. Overall, the transition of raw kraft lignin to foamed 15% PPLF resulted in enhanced thermal stability with less moisture retention, fewer thermal transitions, and more resistance to degradation. These results emphasize the role of PP addition on the foam’s resistance to heat-induced degradation, making it a durable thermal insulation alternative for high-temperature applications.

**Figure 2:**
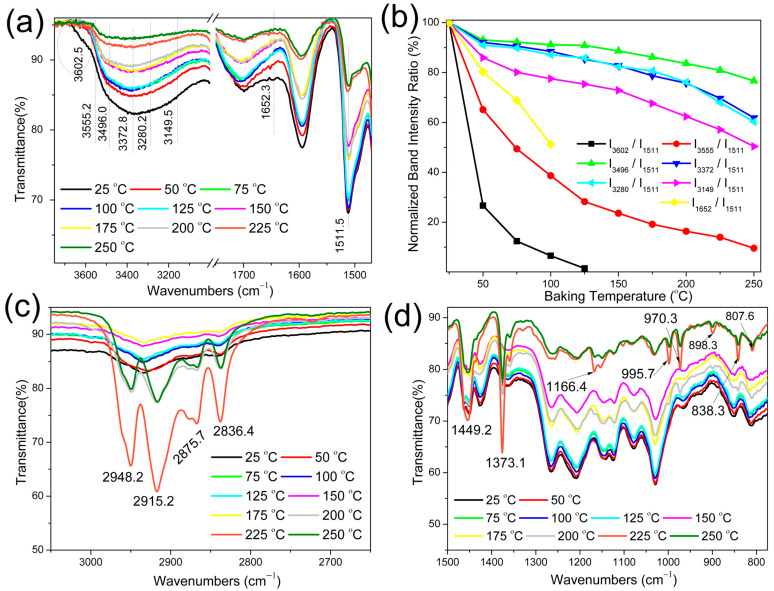
FTIR spectra of PPL4 (15% PP) treated at different temperatures: (**a**) FTIR spectra, (**b**) normalization of intensity ratios of adsorbed water and –OH group bands to the aromatic skeleton band (1511.5 cm^−1^), (**c**,**d**) enlarged FTIR spectra with PP-related bands.

To evaluate the treatment temperature effect, the PPL4 (15% PP) sample was heated to different temperatures, and the FTIR spectra were recorded and compared in Figure 2. Figure 2a showed the FTIR bands related to adsorbed water and the hydroxyl groups of the kraft lignin. In Figure 2b, the intensities of the bands related to the adsorbed water (3602.5, 3555.2, and 1652.3 cm^−1^) attenuated significantly with the increase in the heating temperature. The band intensities were reduced in the order of I_3602.5_ > I_3555.2_ > I_1652.3_ > I_3149.5_ > I_3280.2_ > I_3372.8_ > I_3496.0_, indicating that the adsorbed water species were relatively unstable compared to the chemically bound hydroxyl groups. The FTIR bands of PP are listed in Table S2 and identified in Figure 2c,d [21,22,23]. PP bands were not observed in the FTIR spectra when the 15% PPL sample was heated only to 50–175 °C. However, these bands became detectable in samples heated to 200, 225, and 250 °C, suggesting that PP begins to melt and flow at these elevated temperatures, thereby improving its distribution within the lignin matrix.

### 3.2. Characteristics of Rigid Lignofoams (PPLFs)

#### 3.2.1. Foam Morphology

Representative optical microscopy images of PPLFs are shown in Figure S2. The brown lignin can be clearly seen with the PP (white) distributed throughout the samples PPLF2-6 (Figure S2b–f). PP was added as 0.1–1 mm particles in the PPLs and clearly melted during the foaming process. The PP film can be distinguished from lignin, which forms the main body of the foam, implying limited miscibility while still contributing to structural enhancement. The PP was extended along with the direction of void cellular structures and distributed uniformly in the 3D foam structure. This was in agreement with the detection of PP FTIR bands in 15% PPLs heated over 200 °C in Figure 2.

To further examine the distribution of PP in the lignin matrix before and after foaming, optical microscopy images were captured for both unfoamed PPLs and foamed 15% PPLF (Figures S3 and S4). In the unfoamed preform, the cold-pressed kraft lignin only appears homogeneous and smooth, while in the 15% PPL sample, light-colored PP domains are easily outlined, reflecting its discrete dispersion within the lignin matrix (Figure S3). After foaming, kraft lignin foams exhibit a nonuniform, loosely packed porous structure, whereas PP-containing lignofoams (15% PPLF) develop more defined, interconnected pores with PP redistributed along the pore walls, as shown in Figure S4. This suggests that the PP melts and moves during foaming, stabilizing and reinforcing the foam structure.

**Figure 3:**
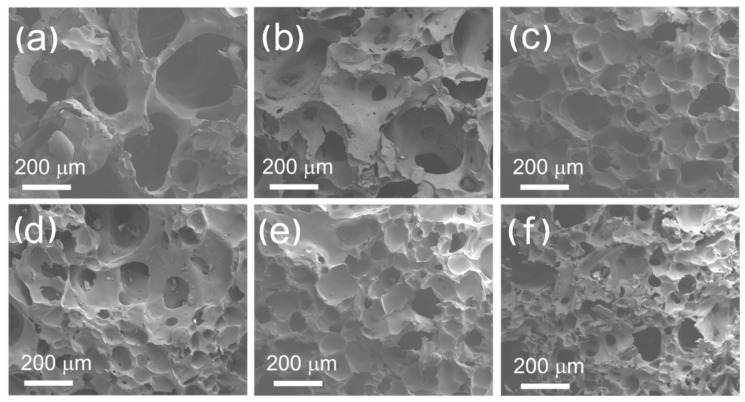
SEM images of PPLF with different PP contents: (**a**) 0%, (**b**) 5%, (**c**) 10%, (**d**) 15%, (**e**) 20%, and (**f**) 30%.

SEM images of PPLFs prepared from various PP contents are shown in Figure 3. The foams appear to exhibit at least a partially open-cell structure, which is presumably because the expanding steam ruptured some of the thin walls of softened lignin between bubbles. This open-cell structure is discussed in more detail in the next section. Figure 3a shows a nonuniform, porous structure for PPLF1 (0% PP). However, lignofoams prepared with 5–30% PP have more uniform distributions and smaller pores (Figure 3b–f).

**Figure 4:**
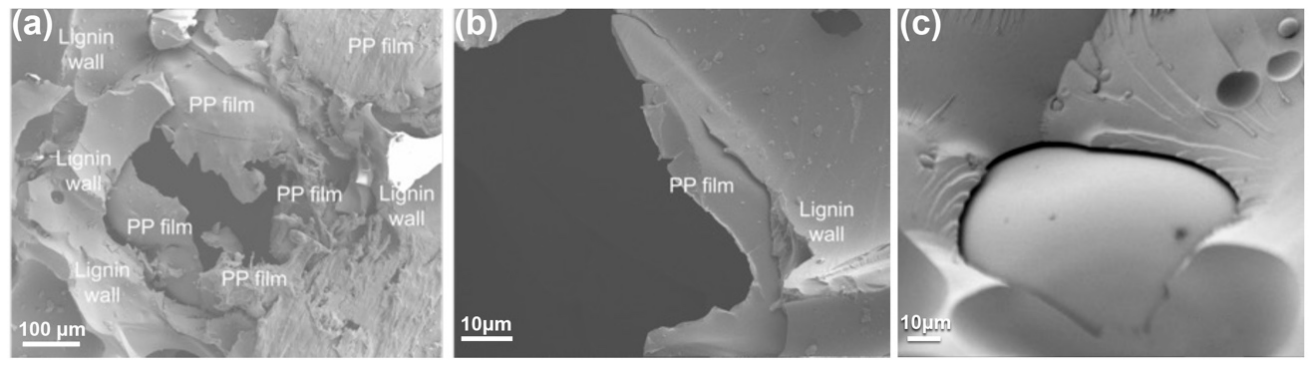
SEM images of PPLF. (**a**,**b**) shows void cell wall of PPLF4 (15% PP) coated with a layer of PP film, (**c**) shows the cell sealed by PP film during foaming.

To further investigate the interaction between lignin and PP in the cellular structure, SEM was used to examine the PP–lignin interface. Micrographs with higher magnification were taken of the fracture surface of the PPLF4 sample (15% PP). Figure 4a,b show that a relatively uniform PP film coating formed on internal pore surfaces. Based on the cross-sectional image, the coating thickness at the edge of the film was about 1–2 µm. Figure 4c clearly shows an interfacial gap between the PP and lignin, which is likely due to their limited miscibility. Despite this, the PP forms a continuous film that coats the pore surfaces and contributes to foam integrity.

#### 3.2.2. Porous Structure

The lignofoam yield, bulk density, porosity, and open-cell rate are listed in Table 1. The foam yield ranged from 93.2 to 95.1 wt% when the PP content increased from 0 to 30 wt%. Since PP is thermally stable over the temperature range investigated, the blowing agents for the self-expanding lignofoams must evolve from the lignin. The TGA results (Figure 1c and Table S1) indicated the evaporation of adsorbed moisture, steam generated from dehydration reactions in kraft lignin dominated the weight loss, and foaming occurred before 240 °C. The increasing foam yield with higher PP content is due to lower weight loss of moisture. The bulk densities of the foams PPLF1~PPLF6 were 0.21, 0.25, 0.28, 0.32, 0.38 and 0.49 g/cm^3^, respectively, showing an increasing trend when the PP content increased. A higher PP content/lower lignin content corresponded to less moisture content in the foam precursor. It resulted in less steam trapped in the softened lignin. Less blowing agent (steam) was unfavorable to the expansion of lignin and caused a less porous structure, leading to an increase in density.

The walls of the pores were coated with melting PP by blowing steam during the foaming process. The porosity and open-cell percentages of PPLFs were calculated based on the bulk density, true density, and true density of pulverized foam [24,25,26]. The porosities are 94.5%, 91.7%, 89.2%, 86.6%, 83,2%, and 78.1% for samples of PPLF1~PPLF6, respectively (Table 1). The porosity of the foam was predominantly controlled by the amount of the blowing agent during foaming. As previously mentioned, most volatiles (blowing agent) could be either adsorbed water or generated from dehydration reactions at elevated temperatures. Consequently, as more PP was added, less blowing agent was present, resulting in lower porosity (Table 1). The percentages of open cells were also reduced with PP addition. This could be due to the lower amounts of blowing agents reducing the number of inter-cellular pathways formed as the foam expands. In addition, the molten PP could seal access to such pathways (Figure 4c).

#### 3.2.3. Compressive Strength and Thermal Conductivity

**Figure 5:**
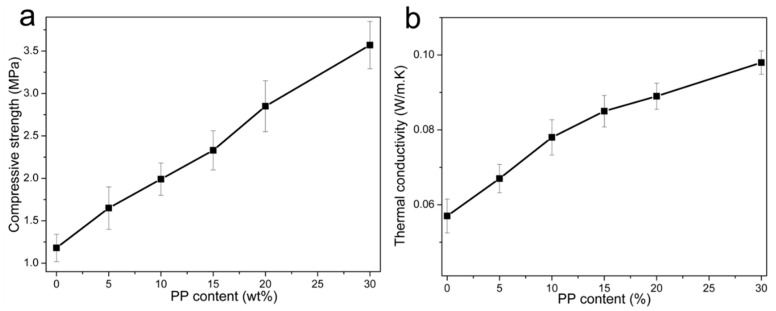
Effect of PP contents on (**a**) compressive strength and (**b**) thermal conductivity of PPLFs.

The mechanical properties of foams depend on the initial preform composition as well as the foam density and microstructure [26]. In Figure 5a, the compressive strength increased from 1.2 to 3.6 MPa with the rising PP content, showing an overall mechanical strength enhancement of up to 200%. Compared to our previous study on the lignofoam based on lignin only [14], PPLF shows higher compressive strength (increased by 9–25%) and porosity (increased by 0.9–3.8%) at the similar densities, which ranges from 0.25 to 0.30 g/cm^3^. Three reasons are postulated for the increased compressive strength during the presence of PP. First, foam density increases with the addition of PP, and the higher the PP content, the greater the density. The compressive strength shows a positive correlation with density, following the same trend as observed in our previous study of lignofoam (lignin only). Secondly, the introduction of PP decreases the number of large cells which can act as defects and reduce strength. PP preserves the pore structure during foaming through its polymer molecular network, maintaining the high porosity of PPLF and preventing pore channel collapse. Another possible reason for improved compressive strength is related to PP’s tendency to coat the porous structure of lignin. While PP is completely immiscible with the lignin and does not chemically bond with it, other mechanisms of adhesion (e.g., mechanical interlocking) may enable PP to help distribute stresses in the lignofoam, mitigating the effects of defects. The strength-to-density (STD) ratios of PPLFs were calculated and listed in Table S3. The results show the STD ratios increasing with increase in PP contents in PPLFs: the STD values are 5.62, 6.60, 7.11, 7.28, 7.50, and 7.29 for PPFL1-PPLF6, respectively. It appears that PPLF5 has the highest STD ratio; i.e., the addition of 20%PP might be the optimized content for the PPLF samples.

The thermal conductivity of a foam material is usually affected by the density, porosity, and fillers added to foams [27]. Figure 5b shows the correlation between the thermal conductivity and different PP contents of the lignofoams. Their thermal conductivity ranged from 0.057 to 0.098 W/mK. The thermal conductivity was directly influenced by the bulk density, which shows a positive correlation [26,28,29]. While adding PP increased the thermal conductivity by more than 72%, the values are still quite below the threshold for materials categorized as thermal insulators (0.1 W/mK) [29]. Moreover, materials with thermal conductivities ranging from 0.03 to 3.0 W/mK are suitable for construction and building materials [30], and those with values below 0.25 W/mK are often used for thermal insulation in those applications. All the PPLF composites proposed in this work fell into that range and therefore have great potential to be used as thermal insulation materials in buildings.

### 3.3. Possible Formation Mechanism

**Scheme 1:**
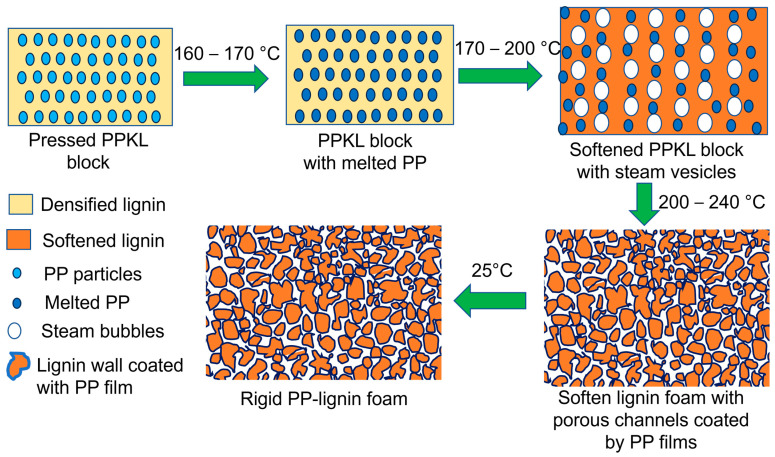
Schematic representation of the foaming mechanism for PP-reinforced lignofoam. Heating induces PP melting and lignin softening, while steam vesicles form and expand the structure. At higher temperatures, the foam stabilizes as PP coats the lignin walls, reinforcing the porous network.

Scheme 1 illustrates a possible foaming mechanism for the PPLFs based on the characterization data as discussed above. The PPL preforms could undergo the following steps: (i) room temperature to 160–170 °C: adsorbed water is released and PP melts, allowing it to diffuse into the lignin structure [31]; (ii) 170–200 °C: lignin softens, hydroxyl groups dehydrate and release moisture in the form of steam (TGA, Figure 1c) [26,28]; (iii) 200–240 °C: more hydroxyl groups dehydrate to form steam that continues to expand the still softening lignin. The expansion rates of PPLFs were calculated through dividing the density of the cold-pressed PPL precursors by the density of the cured PPLFs; the expansion rates were compared to the mass loss of the steam blowing in this step (Table S4). The expansion rates are observed to decrease with the decreasing of steam loss. Increasing steam pressure can rupture cell walls, resulting in an interconnected pore structure. Also, localized differences in steam pressure can aid in distributing the low-viscosity, molten PP, facilitating it to coat the interior of the porous structure. Cooling solidifies the structure, yielding a rigid, open-cell self-expandable foam with a continuous PP film network.

### 3.4. Comparative Analysis of PPLF with Commercial Polyurethane (PU) and Polystyrene (PS) Foams

The physical, thermal, and mechanical properties of PPLFs were compared with established commercial PU, EPS and XPS benchmarks in Table 2. Commercial expanded polystyrene foam (EPS) and extruded polystyrene (XPS) dominate the ultra-low-density foam products with standard grades classified by ASTM C578 ranging from Standard Light (SL) at 12 (EPS) or 19 (XPS) kg/m^3^ to Very High (VH) at 48 kg/m^3^ [32,33,34,35]. The density of commercial rigid polyurethane (PU) foams generally ranges from 12 to 96 kg/m^3^ according to ASTM C1029 [36], while the density of PPLFs in this paper is more dense, ranging from 210 to 490 kg/m^3^. The physical performance of polymer foams is naturally related to their morphology, including the cell size, the cell wall thickness, and the ratio of closed to open cells. Commercial rigid PU foams are engineered to maintain a high closed-cell content (often >90%) to trap low-conductivity blowing gases and provide superior thermal resistance [32,36]. EPS and XPS, in contrast, consist of fused, hollow spherical beads that are 98% air, creating a structure that is chemically inert and lightweight [33,34,35]. The PPLFs are rigid cellular structures with open cells; the open cell rates range from 88.2% to 95.8%. According to the ASTM standards [33,36], the compressive strengths of EPS, XPS and PU foams are listed as 0.035–0.414, 0.104–0.690, and 0.104–0.414 MPa, respectively, while the compressive strengths of PPLFs range from 1.18 to 3.57 MPa due to their high densities. PU, EPS, and XPS foams have exhibited very low thermal conductivities: 0.019–0.030, 0.030–0.040, and 0.025–0.035 W m^−1^ K^−1^ [33,34,35,36]; the PPLFs show relatively high thermal conductivities that range from 0.057 to 0.098 W m^−1^ K^−1^, since they are open-cell structures with higher densities; however, the PPLFs still have potential as the thermal insulation materials in buildings.

## 4. Conclusions

Rigid self-expanding lignofoams (PPLFs) were prepared from unmodified kraft lignin and minor portions (up to 30 wt%) of PP through cold press followed by heat foaming. An interconnected pore structure was formed in PPLF by inherent blowing agents, which primarily came from the vaporization of adsorbed moisture and the condensation of hydroxyl groups during the heat-foaming process. The resulting self-expanding foam had a tunable open-cell structure with inner walls coated by PP films. Adding PP reduced the moisture content, resulting in more uniform pore distributions with porosities of 78.1–94.5%. The lignofoams possessed low densities (0.21–0.49 g/cm^3)^, compressive strengths of 1.18–3.57 MPa, and low thermal conductivities (0.057–0.098 W/mK), showing great potential as thermal insulation in buildings.

## Figures and Tables

**Table 1:** Precursor composition, lignofoam yield, bulk density, porosity, and open-cell percentage of PPLFs.

Sample	Kraft Lignin (wt%)	PP (wt%)	Lignofoam Yield (%)	Bulk Density (g/cm^3^)	Porosity (%)	Open-Cell Percentage (%)
PPLF1	100	0	93.2	0.21 ± 0.02	94.5	95.8
PPLF2	95	5	93.6	0.25 ± 0.01	91.7	94.5
PPLF3	90	10	93.8	0.28 ± 0.01	89.2	93.0
PPLF4	85	15	94.2	0.32 ± 0.02	86.6	91.6
PPLF5	80	20	94.8	0.38 ± 0.02	83.2	90.3
PPLF6	70	30	95.1	0.49 ± 0.01	78.1	88.2

**Table 2:** Property comparison of PPLF vs. commercial polyurethane (PU) and polystyrene (PS) foams.

Material	Density (kg/m^3^)	Cell Morphology	Compressive Strength (MPa)	Thermal Conductivity (W m^−1^ K^−1^)	Source
Expanded polystyrene foam (EPS)	12–48	Fused closed-bead	0.035–0.414	0.030–0.040	[32,33,34,35]
Extruded polystyrene (XPS)	19–48	Fused closed-bead	0.104–0.690	0.025–0.035	[32,33,34,35]
Commercial Rigid PU	12–96	>90% Closed-cell	0.104–0.414	0.019–0.030	[35,36]
Rigid PPLFs	210–490	88.2–95.8% Open-cell	1.18–3.57	0.057–0.098	This work

## Data Availability

Data will be made available on request.

## Supplementary Materials

The following supporting information can be downloaded at https://www.mdpi.com/article/10.3390/polym18050548/s1, Figure S1: TGA, DTG, and DSC analyses of kraft lignin (a–c), and 15% PPLF foam (d–f) over four heating cycles from 30 to 240 °C; Figure S2: Optical images of PPLF samples with different PP contents: (a) 0%, (b) 5%, (c) 10%, (d) 15%, (e) 20%, and (f) 30%.; Figure S3: Optical images of the cross-sections of the cold pressed (a) kraft lignin, and (b) 15% PPL samples.; Figure S4: Optical images of the cross-sections of the foamed (a) kraft lignin, and (b) 15% PPLF samples at 240 °C; Table S1: Temperature ranges, peak temperatures, and weight losses of PPL samples during TGA process between 30 °C and 240 °C; Table S2: FTIR band assignments for polypropylene in PPL samples; Table S3: Density–strength relationship of PPLFs; Table S4: Expansion rates of PPLFs and mass loss of the steam blowing step.
