# Supplementary material for: Eco-Fabrication of Rigid Lignofoams with Porous Cellular Channels Coated by Polypropylene Films for Thermal Insulation Materials
Source: Polymers (Basel). 2026 Feb 25;18(5):548. doi: 10.3390/polym18050548 (PMC12986854; doi:10.3390/polym18050548)
Supplement: Supplementary file 1 [file polymers-18-00548-s001.zip › polymers-4158944-supplementary.pdf]

Supplementary Information

Eco-fabrication of Rigid Lignofoams with Porous Cellular Channels Coated by Polypropylene Films for Thermal Insulation Materials

Numbers of Page: 10

Number of Table: 4

Number of Figure: 4

Calculation of porosity and open cell percentages

Porosity refers to the total volume of voids (both open and closed pores) within a material relative to its total volume. It represents the overall fraction of empty space in the foam structure.

$$\text{Porosity (\%)} = \left(\frac{Volume\ of\ Open\ Cells + Volume\ of\ Closed\ Cells}{Total\ Volume\ of\ Foam}\right) \times\ 100 \qquad \text{(S1)}$$

Open Cell refers to the proportion of those voids that are interconnected (i.e., open to the exterior or other pores), as opposed to isolated, closed cells.

$$\text{Open Cell Percentage (\%)} = \left(\frac{Volume\ of\ Open\ Cells}{Volume\ of\ Open\ Cells\ + Volume\ of\ Closed\ Cells}\right) \times\ 100 \qquad \text{(S2)}$$

Table S1. Temperature ranges, peak temperatures, and weight losses of PPL samples during TGA process between 30 °C and 240 °C.

**Samples**	**Step 1**			**Step 2**			**Step 3**	
	Range (°C)	Peak (°C)	Weight loss (%)	Range (°C)	Peak (°C)	Weight loss (%)	Range (°C)	Weight loss (%)
PPL1	30-146.1	94.4	4.06	146.1-178.4	156.9	0.42	178.4-240	2.31
PPL2	30-140.5	91.8	3.99	140.5-178.8	164.8	0.46	178.8-240	2.01
PPL3	30-137	86.6	3.81	137.0-184.4	165.6	0.58	184.4-240	1.89
PPL4	30-132.3	82.3	3.44	132.3-187.8	165.9	0.64	187.8-240	1.73
PPL5	30-126.1	81.8	2.94	124.1-189.2	166.7	0.66	189.2-240	1.61
PPL6	30-125.8	80.6	2.83	125.8-192.8	166.7	0.66	192.8-240	1.48

Table S2. FTIR band assignments for polypropylene in PPL samples.

**Wave number (cm^{-1})**	**Vibration type**	**Assignment**
807.6	Stretching	C–C
838.3	Rocking	C–H
898.3	Rocking	C–H
970.2	Rocking	CH_3
	Stretching	C–C
995.7	Rocking	CH_3
	Stretching	C–C
1166.4	Wagging	C–H
	Rocking	CH_3
1358.8	Symmetrical bending	CH_3
1373.1	Symmetrical bending	CH_3
1449.2	Symmetrical bending	CH_3
2836.4	Symmetric stretching	CH_2
2875.7	Symmetric stretching	CH_3
2915.2	Asymmetrical stretching	CH_2
2948.2	Asymmetrical stretching	CH_3

Table S3 Density–strength relationship of PPLFs

Sample	Bulk density (g/cm^3)	Compressive strength (Mpa)	Strength-to-density ratio ($MPa/(g/cm^3)$)
PPL1	0.21	1.18	5.62
PPL2	0.25	1.65	6.60
PPL3	0.28	1.99	7.11
PPL4	0.32	2.33	7.28
PPL5	0.38	2.85	7.50
PPL6	0.49	3.57	7.29

Table S4. Expansion rates of PPLFs and mass loss of the steam blowing step.

Bulk density of Cold-pressed PPL (g/cm3)	Bulk density of PPLF (g/cm3)	Expansion rate	Mass loss of the steam blowing step (%)
0.703	0.21	3.35	2.31
0.721	0.25	2.88	2.01
0.739	0.28	2.64	1.89
0.758	0.32	2.37	1.73
0.779	0.38	2.05	1.61
0.823	0.49	1.68	1.48

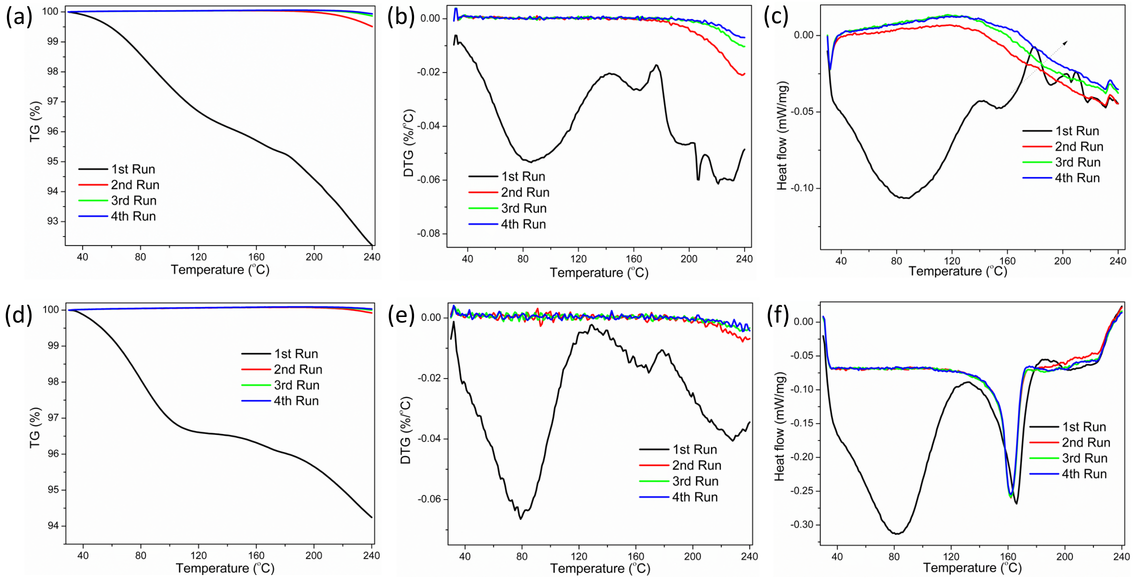

Figure S1. TGA, DTG, and DSC analyses of kraft lignin (a–c), and 15% PPLF foam (d–f) over four heating cycles from 30 to 240 °C.

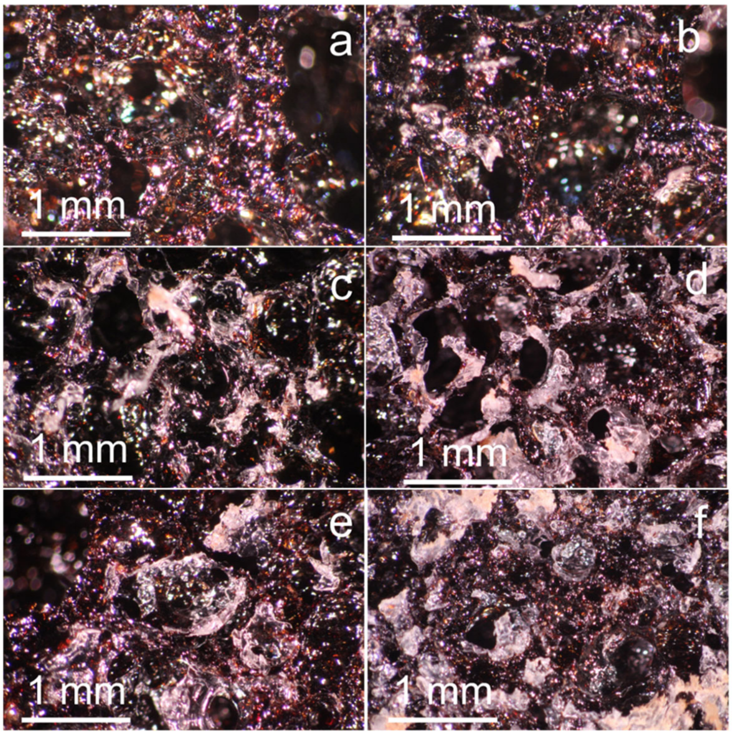

Figure S2. Optical images of PPLF samples with different PP contents: (a) 0 %, (b) 5%, (c) 10%, (d) 15%, (e) 20%, and (f) 30%.

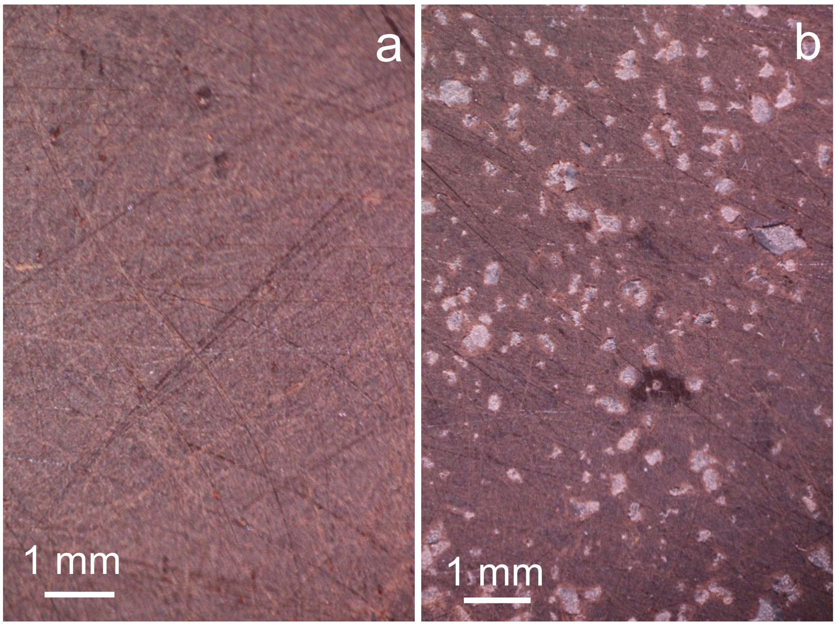

Figure S3. Optical images of the cross-sections of the cold pressed (a) kraft lignin, and (b) 15% PPL samples.

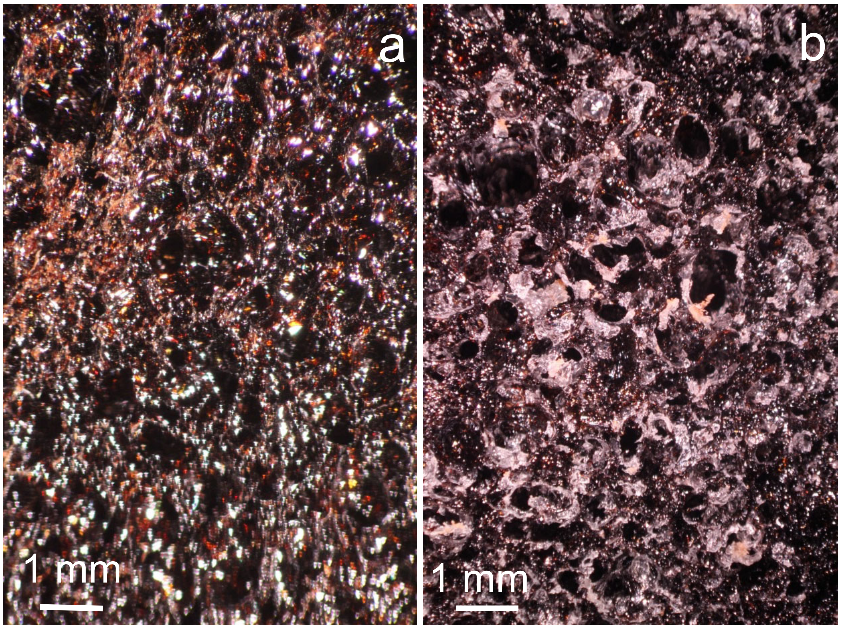

Figure S4. Optical images of the cross-sections of the foamed (a) kraft lignin, and (b) 15% PPLF samples at 240 °C. It should be that the white spots observed in panel (a) are due to the reflection of light from the smooth surface of the lignin foam, not indicative of pore structures.
